# Isolated Bulbar Conjunctival Kaposi's Sarcoma as a Primary Presentation of AIDS: A Case Report

**DOI:** 10.1155/2013/469195

**Published:** 2013-07-01

**Authors:** Sofia Maia, Miguel Gomes, Luís Oliveira, Paulo Torres

**Affiliations:** Departamento de Oftalmologia, Hospital de Santo António, Centro Hospitalar do Porto, Largo Professor Abel Salazar 4099-001 Porto, Portugal

## Abstract

Kaposi's sarcoma (KS) is a malignant vascular tumor, caused by the human herpesvirus 8. It is one of the commonest tumors in human immunodeficiency virus (HIV) patients and not uncommonly the first manifestation of acquired immunodeficiency syndrome (AIDS). *Case*. We present a case of an isolated bulbar conjunctival KS on a 43-year-old HIV positive male, with no other lesions. Excision and cryotherapy were performed, and the patient remains free of lesions to date. *Conclusion*. Isolated bulbar conjunctival KP is an unusual site for its initial presentation and must be kept in mind in HIV positive patients.

## 1. Introduction

 Ocular manifestations of acquired immunodeficiency syndrome (AIDS) are varied, affecting almost all structures of the eye. The posterior segment is mainly affected; however, less frequently ocular adnexa, anterior segment, orbit, and neuroophthalmic lesions can also occur. Human immunodeficiency virus- (HIV-) associated disease occurs in 50–90% of patients in the course of their illness [1].

Kaposi's sarcoma (KS) is the most common lesion of the anterior segment in these patients [2]. It is an AIDS defining illness and affects about 30% of the patients with HIV [2]. It may indicate worsening of the underlying disorder appearing in patients with CD4 count <500 cels/*μ* [1].

KS is a malignant neoplasm of the vascular endothelium that can affect the eyelids, the lacrimal sac or gland, the orbit, and bulbar or palpebral conjunctiva. Isolated KS of the conjunctiva was first reported in 1967 [3]. If it affects the conjunctiva, it appears as a bright red fleshy to violaceous nodular mass, commonly seen in the fornix [2, 4]. 

We report a clinical case of a patient with an isolated conjunctival KS and its treatment.

## 2. Case Report

A 43-year-old HIV positive male since August 2010 presented with a three-month history (since December 2010) of a growing mass in the bulbar conjunctiva of the right eye. The patient had previously undergone right eye cataract extraction with insertion of intraocular lens in 1992 in another medical institution.

**Figure 1:**
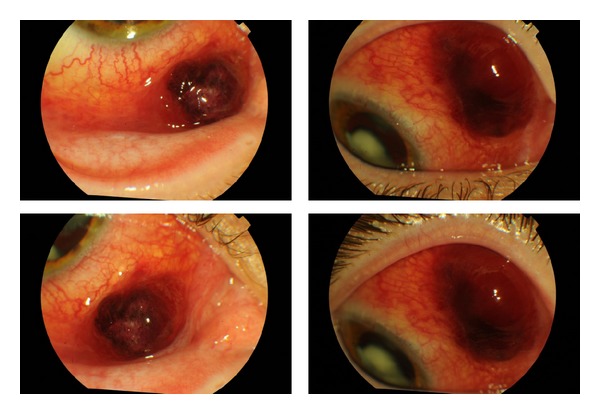
Preoperative biomicroscopy.

On ophthalmic examination, the best corrected visual acuity was 20/25 on his right eye. The biomicroscopy revealed two reddish nodular lesions localized in the superior and inferior nasal bulbar conjunctiva, loosely connected to the underlying tissue (Figure 1). Fundoscopy was normal. The left eye showed no abnormalities. Screening and confirmatory tests were positive for HIV with CD4+ lymphocyte cell count of 154 cells/mm^3^.

**Figure 2:**
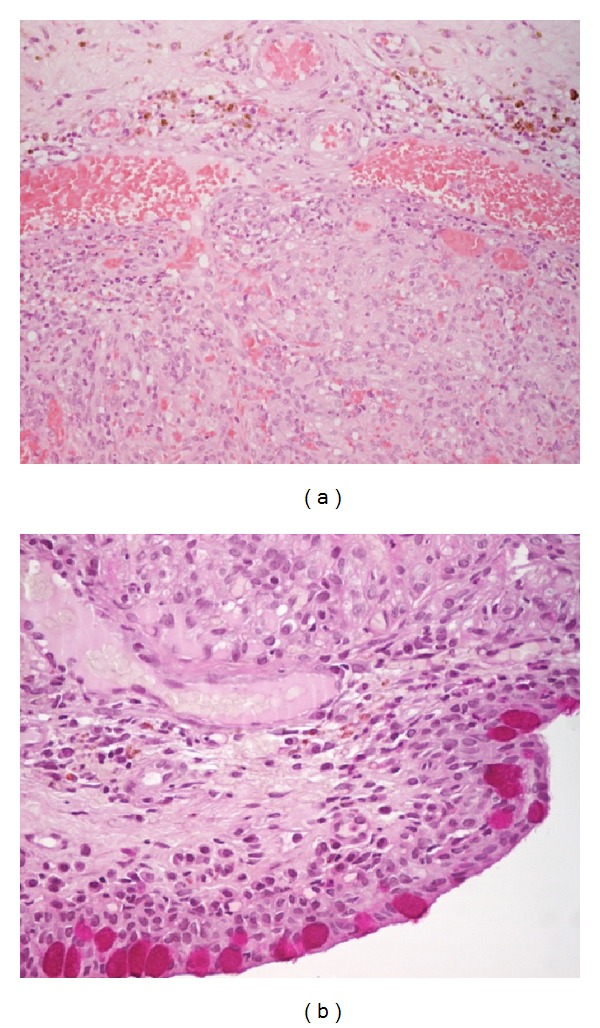
Histopathological finding (a) hematoxylin and eosin stain, (b) peroxide acid-Schiff stain.

**Figure 3:**
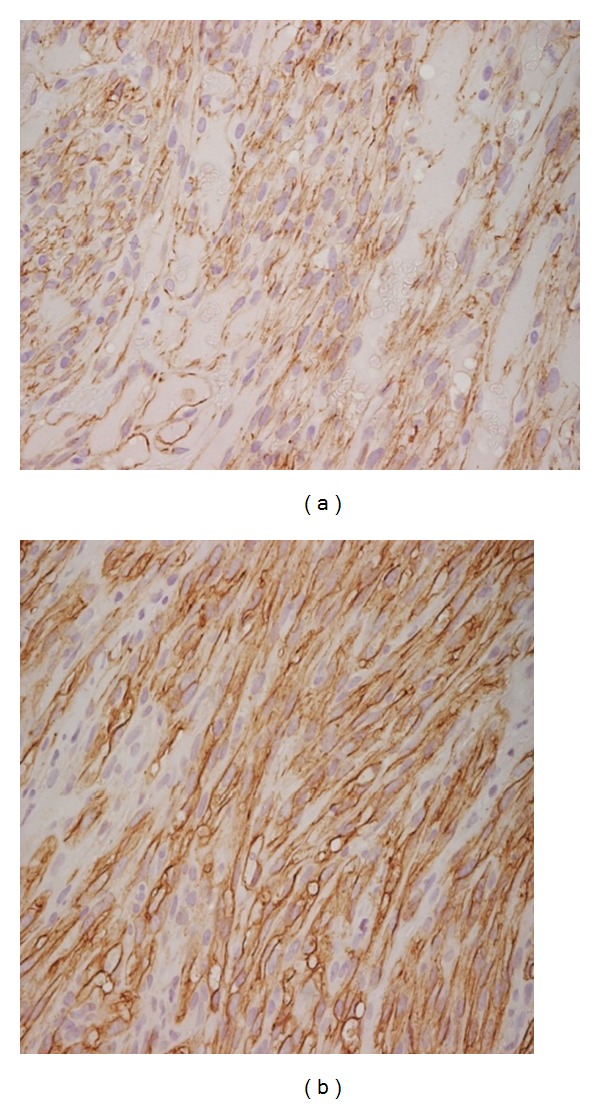
Immunohistochemistry (a) CD31, (b) CD34.

An excisional biopsy and cryotherapy on the conjunctival edges were done on January 2011. Histologic report of the biopsy was consistent with the diagnosis of KS with conjunctival edges free of neoplasm (Figure 2). CD31 and CD34 immunohistochemical stains were positive (Figure 3). CD31 is an endothelial cell marker, CD34 is an important adhesion molecule required for T cells, and both are markers for KS.

**Figure 4:**
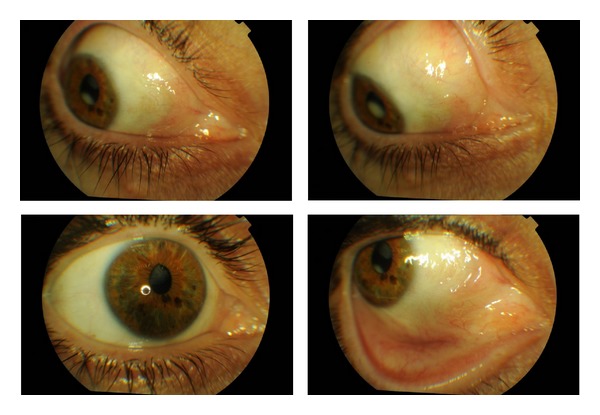
Postoperative biomicroscopy.

The patient initiated standard antiretroviral therapy in January 2011 and remains in followup in the Infectious Diseases Department. Two years later the patient remains with no signs of recurrence (Figure 4).

## 3. Discussion

Improved survival as a result of antiretroviral therapy has led to an increase in systemic and ocular complications. KS is the most common tumor arising in HIV-infected patients. Ophthalmic presentation often occurs in the context of systemic KS, and isolated conjunctival lesions are rare [5–7].

KS may affect many structures of the eye. Involvement of the conjunctiva appears as a flat, reddish lesion, most often localized in the inferior conjunctiva and fornix. It may be misdiagnosed with chronic subconjunctival haemorrhage.

The differential diagnosis includes granuloma pyogenicum, cavernous haemangioma, foreign-body granuloma, malignant melanoma, metastatic tumor, and chronic subconjunctival haemorrhage [7].

Dugel et al. [8] have classified adnexal KS clinically into three stages. In stages 1 and 2, the tumors are patchy and flat (less than 3 mm high), and in stage 3, the tumors are nodular and elevated (more than 3 mm high).

Biopsy with histopathological and immunohistochemistry report has an essential role in the diagnosis.

The type of treatment depends on the size and location of the tumor. KS responds well to treatment by

1. surgical excision: in extensive, large tumors, loosely connected with healthy tissue;
2. radiotherapy: for more extensive lesions;
3. cryotheraphy: for early flat tumors;
4. local injection of cytostatics [9, 10].

This case describes an isolated conjunctival Kaposi's sarcoma as primary presentation of AIDS, which is an unusual location for KS [11].

Complete surgical excision with cryotheraphy on conjunctival edges proved to be effective in our patient, with no evidence of clinical recurrence.

This case emphasizes the importance of considering KS as a possible diagnosis in HIV positive patients. Also, KS can be the first AIDS-defining illness in patients with decreased immunological response.
